# Spectrotemporal Processing in Spectral Tuning Modules of Cat Primary Auditory Cortex

**DOI:** 10.1371/journal.pone.0031537

**Published:** 2012-02-27

**Authors:** Craig A. Atencio, Christoph E. Schreiner

**Affiliations:** Coleman Memorial Laboratory, Department of Otolaryngology-HNS, The UCSF Center for Integrative Neuroscience, University of California San Francisco, San Francisco, California, United States of America; University of Southern California, United States of America

## Abstract

Spectral integration properties show topographical order in cat primary auditory cortex (AI). Along the iso-frequency domain, regions with predominantly narrowly tuned (NT) neurons are segregated from regions with more broadly tuned (BT) neurons, forming distinct processing modules. Despite their prominent spatial segregation, spectrotemporal processing has not been compared for these regions. We identified these NT and BT regions with broad-band ripple stimuli and characterized processing differences between them using both spectrotemporal receptive fields (STRFs) and nonlinear stimulus/firing rate transformations. The durations of STRF excitatory and inhibitory subfields were shorter and the best temporal modulation frequencies were higher for BT neurons than for NT neurons. For NT neurons, the bandwidth of excitatory and inhibitory subfields was matched, whereas for BT neurons it was not. Phase locking and feature selectivity were higher for NT neurons. Properties of the nonlinearities showed only slight differences across the bandwidth modules. These results indicate fundamental differences in spectrotemporal preferences - and thus distinct physiological functions - for neurons in BT and NT spectral integration modules. However, some global processing aspects, such as spectrotemporal interactions and nonlinear input/output behavior, appear to be similar for both neuronal subgroups. The findings suggest that spectral integration modules in AI differ in what specific stimulus aspects are processed, but they are similar in the manner in which stimulus information is processed.

## Introduction

Auditory perception is mediated by functional networks which operate at many scales of organization. At intermediate scales, in the cortical column, spectrotemporal processing is systematically related to layer [1], [2], [3]. However, evaluating networks at larger, intra-areal scales is challenging, since this requires either knowing preexisting network architecture or deriving topographic maps so that connectivity may be related to functional processing. One proxy for preexisting knowledge is to know that a physiological parameter correlates with anatomical connectivity, which then facilitates the evaluation of network function and the consequences of behavioral or physiological changes and manipulations. Such experimental approaches have been exploited in rodent somatosensory cortex for whisker barrels and in cat primary visual cortex for orientation columns [4], [5]. The primary auditory cortex (AI) of the cat also contains an intra-areal network that may be exploited for this purpose. Spectral tuning modules in the cat contain different proportions of narrowly and broadly tuned neurons; regions that contain more narrowly tuned (NT) neurons are anatomically segregated from regions with higher proportions of broadly tuned (BT) neurons [6], [7], [8]. Despite the prominence of this larger scaled AI network, we understand little more than the spectral integration properties of neurons within these spectral tuning modules.

The narrowly and broadly tuned modules are the second most consistently present spatial topography within cat AI [9]. The most fundamental topography is for characteristic frequency (CF), which varies smoothly across the anterior-posterior axis of AI. Along the dorsal-ventral axis, contours have similar CFs and varying spectral tuning [10], [11]. Spectral tuning modules are larger than the traditional column, since they extend over many millimeters, covering CFs from ∼5–20 kHz. AI also contains other spatial representations of less uniformly represented acoustic parameters, such as binaurality, threshold, latency, and intensity tuning, though these do not appear to be related to repeatable, observable ipsilateral network connectivity [12], [13], [14], [15], [16].

Cat AI contains three large-scale spectral integration regions and one that is smaller. The three large tuning regions in AI are: a ventral region, a central region, and a region just dorsal to the central region. These regions are distinguished based on the proportion of NT or BT tuned neurons, with no region completely dominated by only one of the two types of tuning. Thus, ventral AI (vBT), on average, reveals more broadly tuned (BT) cluster responses [17]. Central AI (cNT) is occupied by neuron clusters and single neurons with more narrow tuning (NT) [6], [14], [18]. And the dorsal third of AI (dBT), adjacent to the central narrowly tuned region, is inhabited by a higher proportion of clusters and single neurons that are more broadly-tuned than in the central region than in the central region [17], [19]. Finally, careful, high resolution mapping often reveals a small NT region that is dorsal to the three main tuning regions [6].

The current evidence strongly implies that the NT and BT modules form distinct anatomical networks. Tracer studies showed that the central and dorsal NT regions are anatomically linked [6]; they also showed that the central NT and dorsal BT regions receive inputs from distinct regions in the thalamus [20]. Also, local inhibitory circuits of the central NT module, identified by labeling for the calcium-binding protein parvalbumin, have been shown to remain restricted to this functional region [8]. Last, the presence of multiple narrowly tuned regions is consistent with single-neuron labeling results, which showed that the axons of central AI neurons arborize after projecting over a distance that is nearly the same as that between the central and dorsal narrowly tuned regions [21]. Together, this establishes multiple lines of evidence for NT and BT anatomical networks: multi-unit and single-unit physiology, retrograde cortical labeling, single neuron axon tracing, thalamic projection patterns, and the extent of inhibitory circuits.

The NT and BT modules have been described using pure-tones, though this does not adequately address spectrotemporal processing. To more fully understand acoustic processing, analyses based on broad-band sounds is required [22] and spectrotemporal receptive fields (STRFs) and input/output nonlinearities need to be estimated. The STRF describes the stimulus features that a neuron responds to when challenged with a non-stationary broad-band stimulus. The nonlinearity describes the output of the neuron as a function of the similarity between the stimulus and the STRF, and thus it may capture aspects such as gain, rectification, and saturation. The STRF-nonlinearity model is a compact approach to describing neural function in AI. In this report, we exploit the spatial organization of spectral integration to compare the spectrotemporal processing within AI spectral tuning modules.

## Methods

This study was carried out in strict accordance with the recommendations in the Guide for the Care and Use of Laboratory Animals of the National Institutes of Health. The protocol (AN086113-01B) was approved by the University of California, San Francisco Committee for Animal Research. The electrophysiological recording methods and stimulus design used in this study were previously described in detail [1], [23]. A brief description follows.

### Electrophysiology

Nine young adult cats with clean and otoscopically normal outer and middle ears were sedated with an initial dose of ketamine (22 mg/kg) and acepromazine (0.11 mg/kg), and then anesthetized with pentobarbital sodium (Nembutal, 15–30 mg/kg) for the surgical procedure. The animal's temperature was maintained with a thermostatic heating pad. Bupivicaine was applied to incisions and pressure points. Surgery consisted of a tracheotomy, reflection of the soft tissues of the scalp, craniotomy over AI, and durotomy. After surgery, to maintain an areflexive state, the animal received a continuous infusion of ketamine/diazepam (2–5 mg/kg/hr ketamine, 0.2–0.5 mg/kg/hr diazepam in lactated Ringer solution). All procedures were administered under a protocol approved by the University of California, San Francisco Committee for Animal Research.

With the animal placed inside a sound-shielded anechoic chamber (IAC, Bronx, NY), stimuli were delivered via a closed speaker system to the ear contralateral to the exposed cortex (diaphragms from Stax, Japan). Extracellular recordings were made using multi-channel silicon recording probes, which were provided by the University of Michigan Center for Neural Communication Technology [24]. The probes contained sixteen linearly spaced recording channels, with each channel separated by 150 µm. The contact size of each channel was 177 µm^2^. Having the appropriate impedance for each channel is essential for single-unit recording using the silicon probes. Each channel of the probes had impedances from 2–3 MΩ.

To obtain single neuron responses, neural traces were bandpass filtered between 600 and 6,000 Hz and were digitally recorded with a Cheetah32 A/D system (Neuralynx, Bozeman, MT), at sampling rates between 18,000 and 27,000 Hz. Stimulus-driven neural activity was recorded for approximately 75 minutes at each location. After each experiment, the traces were sorted off-line with a Bayesian spike-sorting algorithm [25]. Most channels of the probe yielded 1–2 well-isolated single units. For example waveforms that may be obtained using this methodology, see Fig. 1 in [26]. All recording locations were in AI, as verified through initial multi-unit mapping and determined by the layout of the tonotopic gradient and bandwidth modules on the crest of the ectosylvian gyrus [9]. For each animal, a digital photo was acquired that contained the posterior ectosylvian, and the anterior ectosylvian sulci. The image was imported into Canvas software (ACD Systems), and subsequent recording positions were marked on the image during the experiment. Recordings were acquired from regions along the dorsal-ventral extent of the 7–25 kHz frequency range in AI [6], [9].

### Stimuli

All neurons were probed with one or two presentations of a 15 or 20 minute dynamic moving ripple stimulus. The ripple stimulus was a temporally varying broadband sound (500–20,000 or 40,000 Hz) composed of approximately 50 sinusoidal carriers per octave, each with randomized phase [27]. The carrier magnitude was modulated by the spectrotemporal envelope. At any given time, the envelope was defined by one spectral and one temporal modulation rate. Spectral modulation rate is defined by the number of spectral peaks per octave across the full bandwidth of the carrier signal. Temporal modulations are defined as the number of peaks per second. Both the spectral and temporal modulation parameters varied randomly and independently over time. Spectral modulation rate varied between 0 and 4 cycles per octave. The temporal modulation rate varied between −40 Hz (resulting in upward sweeps of spectral maxima) and 40 Hz (downward sweep of spectral maxima). Both parameters were statistically independent and unbiased within these ranges. Maximum modulation depth of the spectrotemporal envelope was 40 dB. The mean intensity was set 30–50 dB above the average pure tone threshold in a penetration.

### Analysis

Data analysis was carried out in MATLAB (Mathworks, Natick, MA). For each neuron the reverse correlation method was used to derive the spectrotemporal receptive field (STRF) for all neurons included in the sample [28], [29], [30]. STRFs were thresholded so that only significant features (p<0.01) were included in the analysis [27].

Neuronal spectral and temporal modulation preferences were derived by computing the two-dimensional Fourier transform of each STRF. The FFT is a function of temporal (cycles/s) and spectral modulation rate (cycles/octave). The magnitude of this function was folded along the vertical midline (temporal modulation frequency = 0) to obtain the Ripple Transfer Function (RTF). Since the Fourier transform is sensitive to periodicities in the STRF, the RTF reflects the relationship of excitatory (ON) and suppressive (OFF) STRF subfields. Thus, if the sole STRF feature is an excitatory peak, the RTF will tend to be lowpass in both the temporal and the spectral modulation domains. Strong flanking suppression/inhibition in frequency and/or in time will tend to produce RTFs that are bandpass in the spectral and/or temporal domain.

RTFs were used to obtain modulation transfer functions (MTFs). Summing the RTF along the spectral modulation axis yields the temporal modulation transfer function (tMTF), and summing along the temporal modulation axis yields the spectral modulation transfer function (sMTF). MTFs were classified as bandpass if, after identifying the peak in the MTF, values at lower and higher modulation rates decreased by at least 3 dB. If there was no such decrease for low modulation rates the MTF was classified as lowpass. Highpass MTFs were not encountered. Best modulation frequency for bandpass MTFs was the frequency corresponding to the peak value in the MTF. For lowpass MTFs, the best modulation frequency was defined as the average between zero modulation frequency and the 3 dB high side cutoff. This definition provides a value directly comparable to the estimate for bandpass filters. MTF width for bandpass MTFs was defined as the difference between the high and low 3 dB cutoff values, while for lowpass MTFs the width was the difference between the high side 3 dB cutoff rate and the zero modulation rate.

The independence of the spectral and temporal response properties captured by the STRF, i.e. the spectral-temporal separability, was determined by performing singular value decomposition [3]. Using the decomposition, the separability index was defined as 
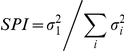
, where 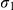 is the largest singular value. The SPI, which ranges between 0 and 1, describes how well the STRF may be described by a pair of 1D functions: one a function of time and the other a function of frequency, with values near 0 corresponding to an STRF for which time and frequency may be dissociated.

Using previously described methodologies, we computed a phase-locking index (PLI) for each neuron using the relation 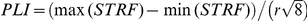, where max(STRF) and min(STRF) are the maximum and minimum values in the STRF, and *r* is the average firing rate [27]. Dividing by *r* and the square root of 8 allows the PLI to range from 0 (not phase locked) to 1 (precisely phase locked). Here phase-locking refers to how well the spikes align to different parts of the ripple stimulus. If the spikes always align to ripple stimulus values that have large magnitudes, then the PLI will be closer to 1, since the difference between the maximum and minimum will be great. When spikes are not as precisely aligned, the maximum in the STRF will decrease, and thus the PLI will decrease in value.

To determine the stimulus selectivity of each neuron we calculated a feature selectivity index (FSI) for each neuron [27], [31]. For each spike generated by the neuron, the ripple envelope that preceded the spike was captured and correlated with the neuron's STRF. The similarity index, SI, is formally defined as 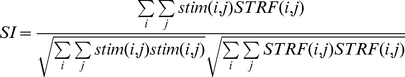
where *stim* and *STRF* are matrices that represent the stimulus segment preceding a spike, and the receptive field of the neuron, respectively, and *i* and *j* range over the number of rows and columns in the STRF. The SI ranges between +1 and −1, and is a measure of the spectrotemporal correlation between the stimulus and the STRF.

A similarity index value was calculated for each action potential, forming a SI probability distribution, 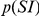, of the driven activity. Using a spike train of similar length but from random spikes [31], [32] we calculated SIs from the neuron's STRF and formed a probability distribution, 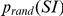, for a random selection of stimulus segments. For each SI probability distribution the cumulative distribution function was then calculated according to
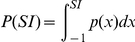
The difference between the random and driven spike trains was quantified by obtaining the areas, *A* and *A_rand_*, under each cumulative distribution function, from which we then calculated the FSI as 
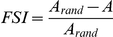
FSI values vary between 0 and 1, where 0 corresponds to similar distributions for 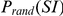 and 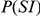, i.e., a neuron that responds indiscriminately to stimulus segments, and 1 corresponds to a neuron that is responsive to a very restricted and fixed range of stimulus features.

For each STRF, we computed the nonlinear input/output function that related the stimulus to the probability of spike occurrence [33]. The following steps were used to calculate the nonlinearities. (1) Each ripple stimulus segment, *s*, that elicited a spike, was correlated with the STRF by projecting it onto the STRF via the inner product 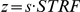. These projections, or stimulus-filter similarities, characterize the probability distribution 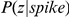. (2) We then projected a large number of randomly-selected stimulus segments onto the STRF, and formed the prior stimulus distribution, 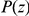. (3) The mean and standard deviation of 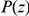, μ and σ, were then calculated. (4) 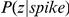 and 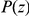 were transformed to units of standard deviation by using 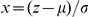, to obtain the distributions 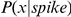 and 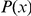. The *x* values here are not similarity index values since the *x* values are normalized differently. Because of the transformation, the values of *x* are now in units of standard deviation (SD). (5) The nonlinearity for the STRF was then computed as 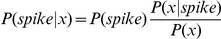, where 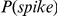 is the average firing rate of the neuron. Thus, the nonlinearity describes the likelihood of a spike given the similarity between the STRF and the stimulus. High *x* values indicate STRF-stimulus correlations that would not be expected from a randomly spiking neuron, while values near 0 would be expected if the neuron fired indiscriminately. Thus, if nonlinearity values increase as the *x* values increase, then greater similarity between the STRF and the stimulus results in greater firing rates.

## Results

We studied the spectrotemporal processing properties of neurons in different topographic regions of AI. We recorded from neurons in the central narrowly tuned region of AI, and in regions ventral and dorsal to this central region [6], [9], [14]. The majority of characteristic frequencies (CFs) were between 7 and 25 kHz, a range that shows the strongest spatial segregation of broadly and narrowly tuned neurons along the iso-frequency domain [9]. To examine spectrotemporal processing, we recorded the responses of AI neurons while presenting a dynamic ripple stimulus that was approximately 40 dB SPL above the pure-tone threshold of each neuron. We then estimated the spectrotemporal receptive field (STRF) of each neuron by calculating the average spectrotemporal stimulus envelope preceding an action potential (the spike-triggered average, STA). In this report, we used the STRF as an assay for spectrotemporal processing.

### STRF Classification

We recorded from 1063 neurons and used the STRF to identify NT and BT neurons. From the STRF, we computed the spectral tuning, Q, by dividing the CF by the excitatory bandwidth of the neuron (Q = Characteristic frequency/bandwidth = CF/BW). The CF was identified as the peak in the excitatory portion of the STRF, and the bandwidth was the width of the STRF when the excitatory portion of the STRF was 10% of the maximum STRF value. We classified a neuron as BT if the STRF-based Q value was less than 1.5. A cell with a Q value above 3.5 was designated as NT.

**Figure 1:**
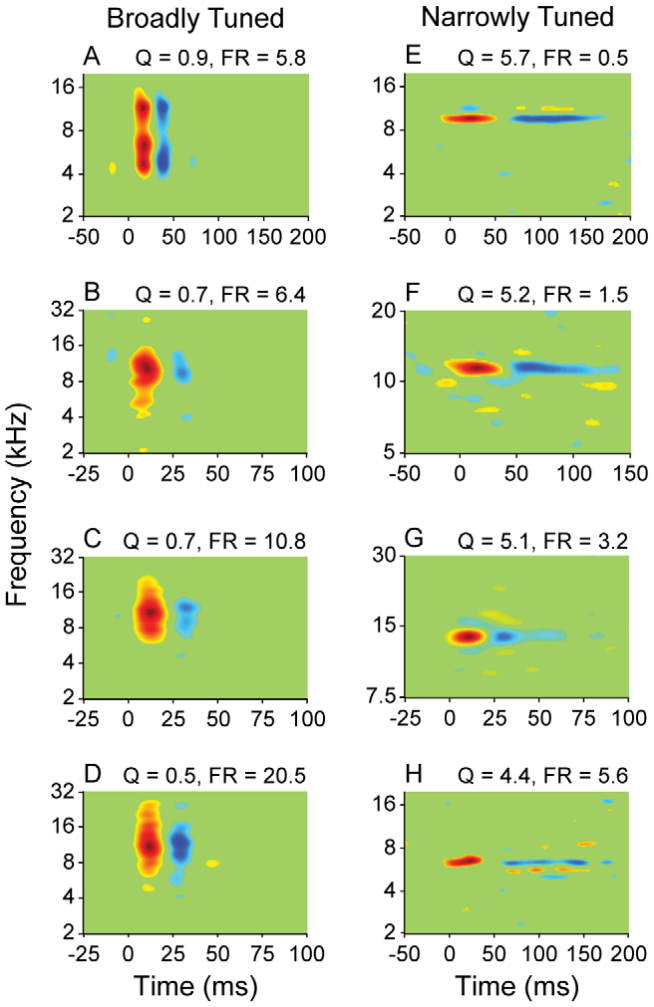
STRFs of broadly and narrowly tuned AI neurons. (A–D) Broadly tuned neurons. (E–H) Narrowly tuned neurons. Q: quality factor, which is the spectral tuning metric. FR: firing rate.

When we divided neurons into NT and BT classes, the STRFs of each class diverged beyond that expected for mere spectral bandwidth (Fig. 1A–D: BT STRFs; Fig. 1E–H: NT STRFs). NT neurons had STRFs with diverse shapes, though one consistent characteristic was a well-defined excitatory subfield preceding an inhibitory subfield (Fig. 1E–H; excitatory subfield: red; inhibitory subfield: blue). The excitatory subfield usually had a shorter duration than the inhibitory subfield, and the spectral bandwidth of the main excitatory subfield was similar to the bandwidth for the inhibitory subfield. This was not the case for BT neurons (Fig. 1A–D). Also in contrast to NT neurons, most BT neurons had excitatory subfields with short temporal durations. The inhibitory subfield of BT neurons was of similar temporal duration to the excitatory one, though the inhibitory bandwidth often did not match the excitatory bandwidth.

### Recording Locations

**Figure 2:**
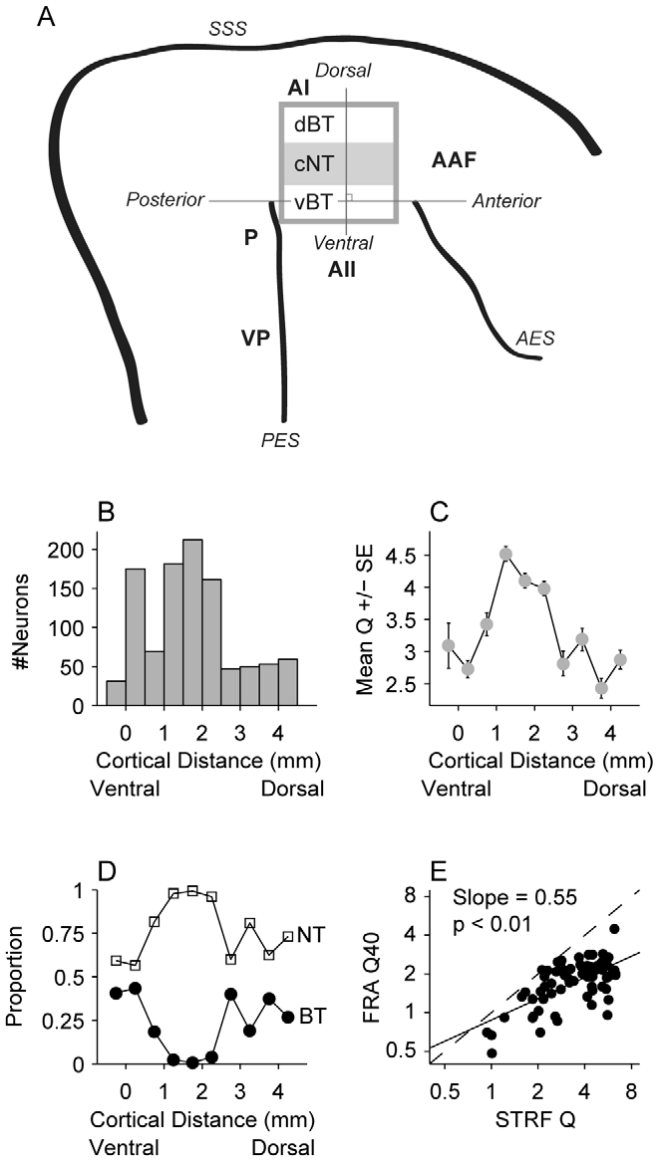
Recording location analysis. For each experiment, recording locations were noted on a digital photo of AI. Locations were estimated along the dorsal-ventral axis of AI by projecting the recording position onto a line that was orthogonal to a line connecting the tips of the anterior and posterior ectosylvian sulci. (A) Schematic of auditory cortex and spectral tuning modules. Rectangle in AI represents the region within AI where spectral tuning modules are present (dBT: dorsal broadband region; cNT: central narrowband region; vBT: ventral broadband region). Dorsal-ventral position of each electrode penetration was calculated by projecting recording sites onto the line that is orthogonal to the line connecting the posterior ectosylvian (PES) and anterior ectosylvian sulcus (AES). SSS: suprasylvian sulcus. AAF: anterior auditory field; AII: secondary auditory field; P: posterior auditory field; VP: Ventral-posterior auditory field. (B) Number of recorded neurons along the dorsal-ventral axis. (C) Mean STRF Q values along the dorsal-ventral axis. Tuning is sharpest in the central region of AI. (D) For broadly tuned (BT) and narrowly tuned (NT) neurons, the proportion of each along the dorsal-ventral axis. BT neurons had STRF Q values < = 1.5, and NT neurons had Q values > = 3.5. Almost no BT neurons are found within the central region of AI. (E) Relation between STRF Q values and frequency response area (FRA) Q40 values. Dashed line represents equality, and solid line represents the best fit (slope = 0.55, p<0.01, t-test).

One of the most striking features of cat AI is the change in the proportion of tuning types along the dorsal-ventral axis (Fig. 2A). The presence of tuning types is not all-or-none; it means that there are higher proportions of either NT or BT neurons in the main regions of AI. We confirmed this using STRFs: neurons were recorded across the dorsal-ventral extent of AI and then collated across animals. We used a multi-step process to combine neurons across animals. First, during each experiment, penetration sites were marked on a digital image of the cortical surface. We then used the image of AI to construct a line that extended from the tip of the posterior ectosylvian sulcus (PES) to the tip of the anterior ectosylvian sulcus (AES). Previous mapping studies have indicated that this line is usually positioned near the ventral border of AI, but not within AII [9]. We next constructed a line that was orthogonal to the line connecting the tips of the sulci (see Fig. 2A for a schematic). Last, we projected each recording site onto the orthogonal line, allowing us to estimate the dorsal-ventral recording position for each neuron.

Significant numbers of neurons were recorded throughout AI, with the greatest yield from the central portion (Fig. 2B). Despite the possible variability of AI relative to sulcal patterns [10], we found that the mean local Q values unambiguously varied across the dorsal-ventral axis, with the highest Q values in the central portion of AI (Fig. 2C). The proportion of narrowly and broadly tuned neurons also varied systematically, and in accord with previous studies [6], [9], [17]: the central region had the greatest proportion of NT neurons and almost no broadly tuned neurons (Fig. 2D). Finally, we compared the STRF Q values to Q40 values obtained with pure tones (Fig. 2E) [9]. The STRF Q values were generally higher than the pure tone Q40 values (slope of best fit line = 0.55, p<0.01, t-test), indicating sharper tuning estimates for STRFs. The causes for this differences are likely related to (a) different criteria used to define the edge of a pure-tone tuning curve versus the excitatory STRF portion, and (b) the stimulus-dependence of receptive fields, in this case the narrowband nature of the pure tones versus the broadband nature of the ripple stimuli. Despite these differences, both pure tone and STRF analyses have now revealed the dorsal-ventral variation of spectral tuning, further indicating that the spectral integration modules are a fundamental feature of cat AI.

In the presentation that follows, NT neurons were grouped together for statistical analysis if they were located in the central narrowly tuned region of AI (Fig. 2B,C,D; 0.65 mm–2.85 mm, N = 393). For broadly tuned neurons, we did not find statistical differences among the various tested characteristics when we compared neurons in ventral AI to those in dorsal AI. Thus, all broadly tuned neurons were included in the BT class (N = 123).

### Response Strength

BT and NT neurons differed in their response strength to the ripple stimulus, with BT neurons having a higher firing rate (Fig. 1, insets). In response to the ripple stimulus, BT neuron discharge rates often exceeded 15 spikes/s (BT firing rate (sp/s): median = 7.4, median absolute deviation (MAD) = 5.5). The firing rates of NT neurons were significantly less (NT firing rate (sp/s): median/MAD: 3.3/2.4, p<0.001, Rank-sum test).

### Quantifying the Duration and Bandwidth of STRF Subfields

**Figure 3:**
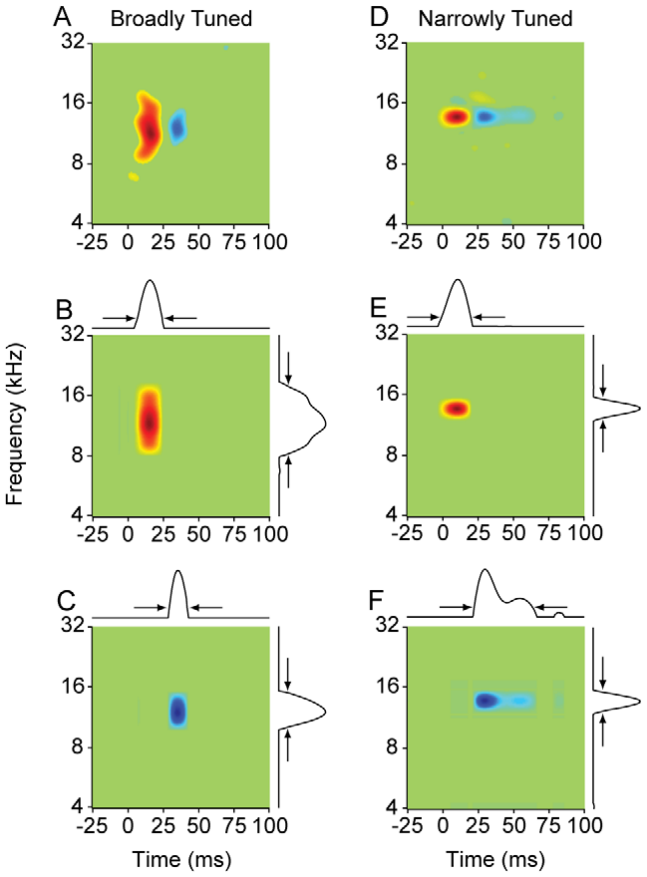
Method for determining the temporal and spectral extent of excitatory and inhibitory STRF subfields. Each column represents one neuron (A–C: broadly tuned; D–F: narrowly tuned). Singular value decomposition (SVD) was performed on either the excitatory (B,E) or inhibitory subfield (C,F). Time and frequency 1D marginals are shown above, and to the right, of each separable component in B, C, E, F. The first separable component from the SVD analysis was used to extract the subfield width at 10% of the peak value (arrows).

Using the STRF, we quantified excitation and inhibition for all neurons by estimating the bandwidth and duration of the excitatory and inhibitory STRF subfields. We first decomposed each STRF into its excitatory (firing rate increase, red subfields in Fig. 3A,D) and inhibitory components (firing rate decrease, blue subfields in Fig. 3B,E). We then performed singular value decomposition on the components, and obtained profiles of excitation and inhibition along the frequency and time axes (Fig. 3B,C,E,F; profiles shown as marginal plots). For each STRF, we estimated the duration and bandwidth of excitation and inhibition by determining when the profile values decreased to 10% of the peak value (indicated by arrows in Fig. 3B,C,E,F). The width at the 10% value was then estimated to be the duration or bandwidth.

### Comparison of Excitatory and Inhibitory Subfield Durations and Bandwidths

**Figure 4:**
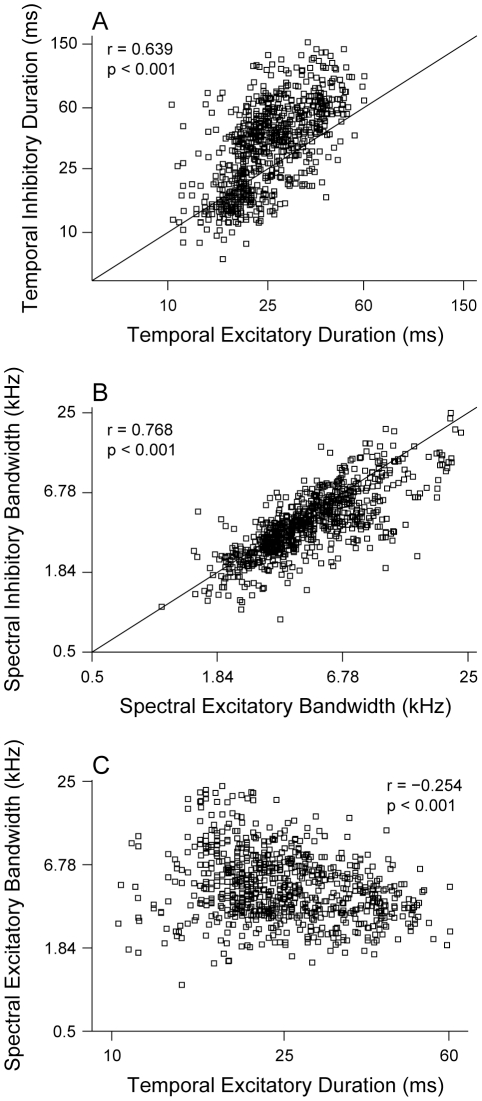
Comparison of temporal and spectral STRF subfield duration and bandwidth. (A) Temporal inhibitory STRF subfield duration versus temporal excitatory subfield duration. Inhibitory duration was longer than excitatory duration (r = 0.639, p<0.001, t-test). (B) Spectral STRF bandwidth for inhibitory versus excitatory subfields. Bandwidth for excitation was generally similar to the bandwidth for inhibition (r = 0.768, p<0.001, t-test). (C) Excitatory STRF subfield bandwidth versus duration. Excitatory bandwidth decreases slightly with increasing duration (r = −0.254, p<0.001, t-test).

Over the entire population of neurons, STRF excitatory and inhibitory subfields were correlated with respect to duration and bandwidth. The duration of the excitatory subfield was strongly correlated with the duration of the inhibitory subfield (Fig. 4A; r = 0.639, p<0.001). The excitatory subfield duration, however, was systematically shorter than the inhibitory subfield duration (Fig. 4A: diagonal line represents identity relationship). The bandwidths of excitatory and inhibitory STRF subfields were also highly correlated (Fig. 4B; r = 0.768, p<0.001). We also found that the bandwidths of excitatory and inhibitory subfields were matched, indicating that, on average, changes in excitatory bandwidth are accompanied by similar changes in inhibitory bandwidth. Last, we found a weaker correlation between excitatory subfield bandwidth and excitatory subfield duration (Fig. 4C; r = −0.254, p<0.001). As the duration of the excitatory subfield increased, the bandwidth of the excitatory subfield decreased. This implies that neurons with the narrowest excitatory tuning have longer-lasting excitation.

**Figure 5:**
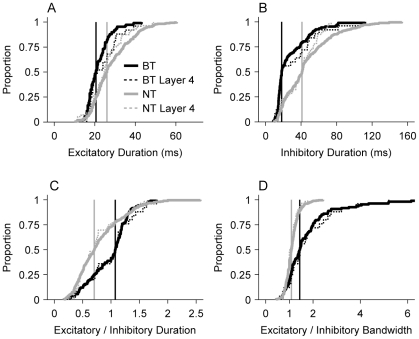
Comparison between BT and NT neuron STRF subfield duration and bandwidth. (A) Cumulative distribution function (CDF) of excitatory subfield durations. Solid lines represent total population data. Vertical lines indicate population medians. Dashed lines represent values exclusively from layer 4 neurons (800–1100 µm cortical depth). NT neurons have significantly longer excitatory subfield durations (p<0.001, KS-test). (B) CDF of inhibitory duration. BT neurons had shorter inhibitory subfields (p<0.001, KS-test). (C) CDF of the ratio of excitatory to inhibitory duration. For BT neurons, the temporal duration of excitation is similar to the duration of inhibition (p<0.001, KS-test). (D) CDF of the ratio of excitatory to inhibitory bandwidth. For BT neurons, excitatory bandwidth was greater than inhibitory bandwidth. For NT neurons, the bandwidths were similar (p<0.001, KS-test).

For BT neurons, the excitatory subfield duration was shorter (BT median/MAD: 20.5/3.4 ms versus NT median/MAD: 26.0/6.5 ms), indicating a lower range of excitatory duration compared to NT neurons (Fig. 5A; p<0.001, KS-test). Additionally, BT neurons had much shorter mean inhibitory subfield durations (BT median/MAD: 18.0/4.5 ms versus NT median/MAD: 41.0/18.5 ms), with BT neuron values significantly lower (Fig. 5B; p<0.001, KS-test).

We also compared the excitatory and inhibitory subfields with respect to the duration and bandwidth of each NT and BT neuron. To determine if subfield durations were similar, we divided the duration of the excitatory subfield by the duration of the inhibitory subfield. Values near 1 indicate that the durations were similar, while values less than 1 indicate that inhibition lasts longer than excitation. For BT neurons, subfield durations were closely matched (median/MAD = 1.07/0.21), while the inhibitory subfields of NT neurons were longer than the excitatory subfield (Fig. 5C; median/MAD = 0.70/0.26). Since the subfields of BT neurons are shorter, this predicts that BT neurons will respond to temporal modulations differently than NT neurons. Thus, BT and NT neurons should differ in how they process the sequential acoustic elements in complex sounds (see below).

We then examined if the bandwidth of STRF excitatory subfields was similar to the bandwidth of inhibitory subfields. By definition, BT and NT neurons had excitatory bandwidths that were significantly different. This does not describe, however, if within each class the bandwidth of excitatory and inhibitory subfields are similar. By dividing the bandwidths of the two subfields, we found that the mean ratios for BT neurons were larger (Fig. 5D; BT median/MAD = 1.44/0.44; NT median/MAD = 1.08/0.15). BT neurons often had excitatory bandwidths 2 to 3 times greater than inhibitory bandwidths. Since the bandwidths of excitatory and inhibitory sequential subfields are not matched, the frequency components of a broadband sound are processed differentially by the receptive fields of BT neurons. This suggests strong differences in the influence of short-term spectral and temporal context on BT and NT neurons.

So far, the population analysis was based on neurons distributed across the full depth of AI. Previously we have demonstrated that cortical receptive fields may show some layer specificity within the columnar organization [1], although the within-column variations appeared smaller than variations across different functional sub-regions in AI as observed in the thalamic input layers. To begin to disambiguate effects of laminar and regional variations we repeated our analysis only for neurons from the thalamic input layer 4 (Fig. 5 A–D, dashed lines). The effects obtained for the whole population and layer 4 were largely similar for the ratios of excitatory/inhibitory duration and bandwidth (Fig. 5C,D) and, slightly less, for the inhibitory duration (Fig. 5B). This suggests that local differences between cortical input and output layers do not affect global differences between functional subregions, the basis of the BT versus NT distinction. However, the excitatory duration (Fig. 5A) showed no difference between NT and BT neurons in the input layer in contrast to the whole population, indicating that excitatory duration is affected by cortico-cortical interactions in the output layers.

### Modulation Processing of AI Neurons

**Figure 6:**
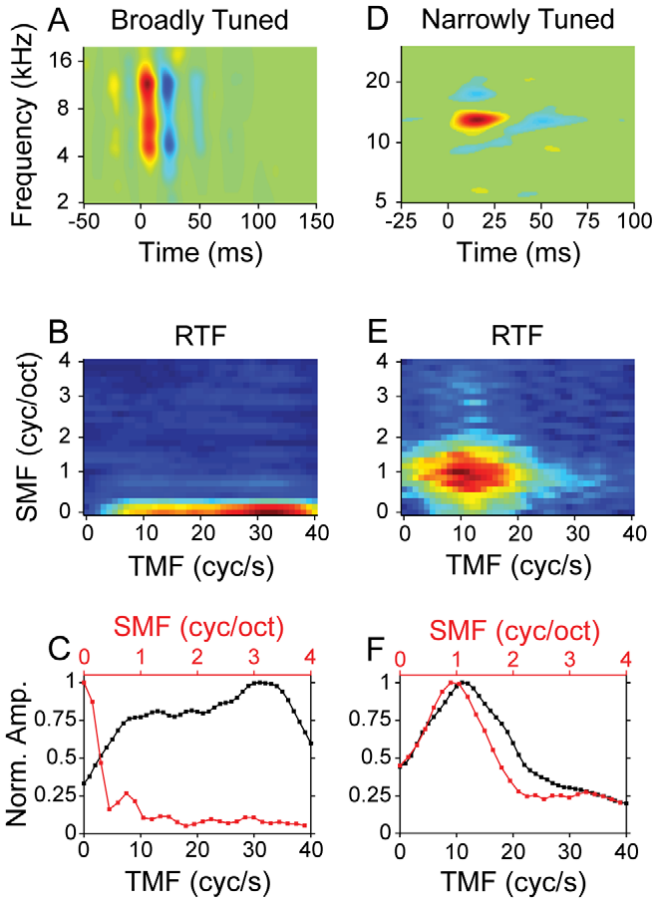
Modulation processing analysis. (A) Broadly tuned neuron STRF. (D) Narrowly tuned neuron STRF. (B,E) Ripple Transfer Functions (RTFs) of the STRFs. (C,F) Temporal (black) and Spectral (red) modulation transfer functions (MTFs) are obtained from the RTF by summing across spectral or temporal modulation frequency, respectively. (C) The BT neuron has bandpass tuning for temporal modulations, and lowpass tuning for spectral modulations. (F) The NT neuron has bandpass tuning for both temporal and spectral modulations.

We next examined how AI neurons process amplitude variations within the temporal and spectral dimensions. A neuron's responses to amplitude variations may be described by modulation transfer functions (MTFs). MTFs describe the response as a function of the periodicity at which the variations occur. We calculated MTFs in a multi-step process. First, we calculated the 2D Fourier transform of each STRF (Fig. 6A,D). The absolute value of the transform is the ripple transfer function (RTF; Fig. 6B: BT neuron RTF; Fig. 6E: NT neuron RTF). The RTF describes STRF energy as a function of modulation frequency. For a NT neuron with inhibitory spectral sidebands, the RTF has energy in a compact region of the modulation parameter space (Fig. 6E). This implies that only a narrow range of spectral and temporal modulation values excite the neuron. From the RTFs we then obtained 1D temporal and spectral MTFs. The MTFs were obtained by summing the RTF across the spectral and temporal modulation axes, respectively, resulting in either the temporal or spectral MTF (tMTF, black; sMTF, red; BT neuron MTFs: Fig. 6C; NT neuron MTFs: Fig. 6F). The MTFs for a NT neuron with spectral and temporal suppression are bandpass (Fig. 6F; best temporal (bTMF) and best spectral (bSMF) modulation frequencies near 10 cycles/second and 1 cycle/octave, respectively).

BT neurons had MTFs that were fundamentally different from NT neurons. BT neurons responded to spectral modulations largely in a lowpass fashion (Fig. 6C), due to the lack of significant inhibitory sidebands. Since BT neurons have shorter excitatory and inhibitory STRF subfields, they followed faster temporal amplitude modulations than NT neurons. Indeed, some neurons followed quite fast temporal modulations, up to 30 cycles per second, which is higher than the mean best temporal modulation frequencies of AI neurons [32], [34], [35].

**Figure 7:**
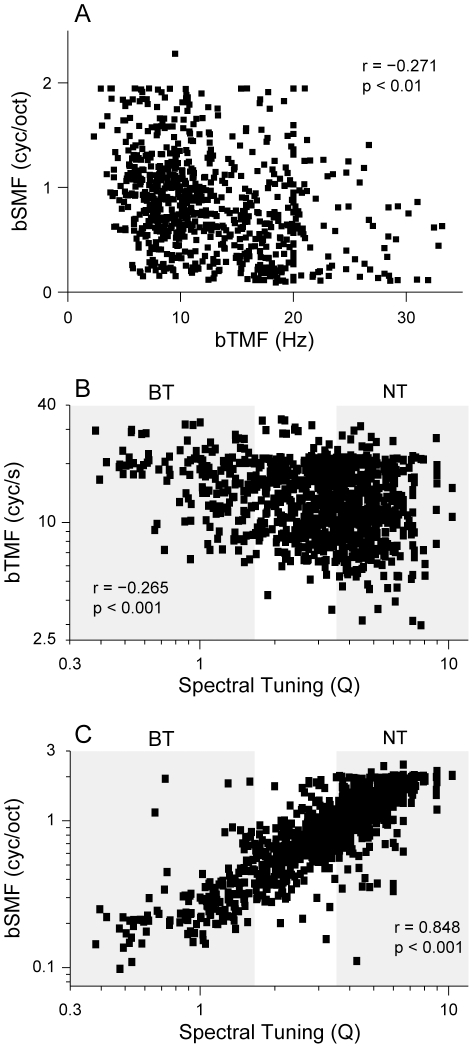
Best modulation frequency. (A) Best spectral modulation frequency (bSMF) versus best temporal modulation frequency (bTMF). bSMF is weakly correlated with bTMF (r = −0.271, p<0.01, t-test). (B) bTMFs decrease as tuning sharpness increases (p = −0.265, p<0.001, t-test). (C) bSMF is highly correlated with spectral tuning (r = 0.848, p<0.001, t-test). Shaded areas in (B,C) indicate Broadly Tuned (BT) and Narrowly Tuned (NT) neurons.

For all recorded neurons, we calculated the best temporal (bTMF) and spectral (bSMF) modulation frequencies from each MTF. bTMF was weakly related to bSMF (Fig. 7A; r = −0.271, p<0.01). Consistent with previous work [32], the correlation between the best modulation frequencies showed large scatter and variability, with the majority of neurons having bTMFs from 8–16 Hz, and bSMFs from 0.5–1 cyc/octave. In this main window of modulation frequencies, there is little correlation between the two parameters, indicating that the strong temporal and spectral modulation processing tradeoffs seen in the auditory midbrain and thalamus [32], [36], [37] are not strongly expressed in AI.

Analyzing the relationship between best modulation frequency and spectral tuning revealed that bTMF trended toward a negative correlation with spectral integration (Fig. 7B; r = −0.265, p<0.001, t-test). bSMF was positively correlated with spectral tuning (Fig. 7C; r = 0.848, p<0.001, t-test). One likely reason for the latter is that the relative spacing of inhibitory spectral sidebands changes depending on the bandwidth of the neuron. As the bandwidth of a neuron increases, the highest spectral modulation frequency the neuron can resolve decreases. Because spectral modulation preference varies with spectral tuning, and since there is already a spectral integration topography present in AI, there may be other, perhaps more local, topographies of spectrotemporal parameters within AI.

**Figure 8:**
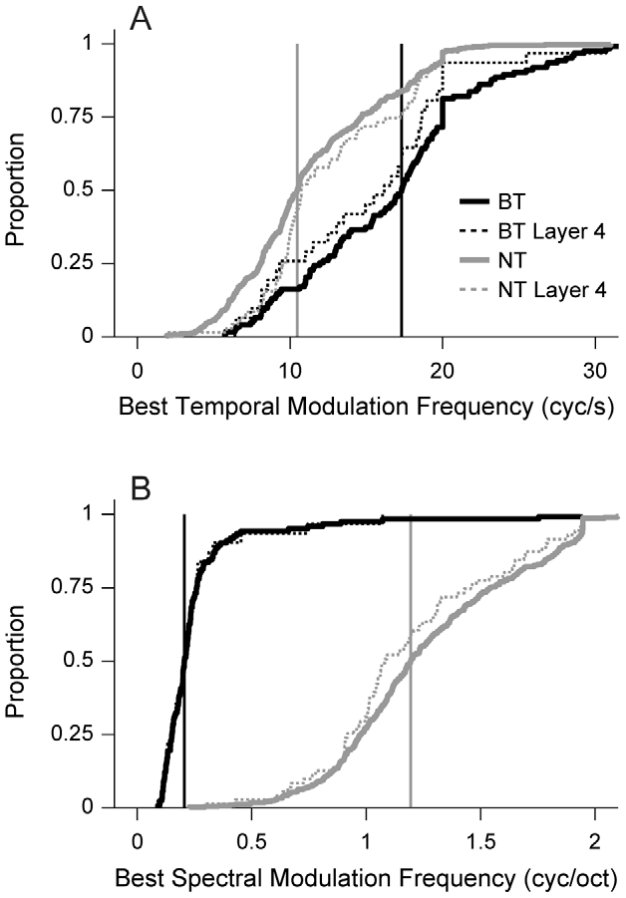
Best modulation frequencies of BT and NT AI neurons. (A) Distribution of best temporal modulation frequencies (bTMFs). Solid lines represent population data. Vertical lines indicate population medians. Dashed lines represent values for neurons in layer 4 (800–1100 µm). BT neurons had higher bTMFs (p<0.001, KS-test). BT neurons have significantly higher bTMFs. (B) Distribution of best spectral modulation frequencies (bSMFs). NT neurons had higher bSMFs (p<0.001, KS-test).

We next compared best modulation frequencies for BT and NT neurons. We found that the bTMFs for BT neurons were significantly higher (Fig. 8A; BT median/MAD = 17.3/4.0; NT median/MAD = 10.5/3.2; p<0.001, Rank-sum test), indicative of better temporal modulation processing (p<0.001, KS-test). In a similar fashion, we also compared the bSMF values (Fig. 8B), and found that NT neurons had significantly higher spectral modulation values (BT median/MAD: 0.21/0.05 cyc/oct; NT median/MAD = 1.19/0.27 cyc/oct). The bSMF result was expected, however, since it follows from the classification scheme we used to obtain the BT and NT neuron classes. Comparing differences between the subpopulations based only on layer 4 neurons (Fig. 8A,B dashed lines) shows no clear layer-based effect.

The shape of the MTF also characterizes how AI neurons respond to dynamic sounds. Though the bTMF describes the preferred presentation rate of acoustic energy, it does not describe how this information is processed. For BT neurons, 63% had lowpass tMTFs, and 37% had bandpass. The higher amount of lowpass MTFs likely reflects the decrease in temporal following inhibition that occurs for many BT neurons (see STRFs in Fig. 1D,E). In contrast, NT neurons have more evenly distributed tMTF shapes (56% bandpass, 44% lowpass), indicative of the stronger sequential suppression in the central region of AI.

To determine if the proportions of MTF shapes were significantly different, we performed a randomization test. For the randomization test, the null hypothesis was that the distribution of bandpass and lowpass MTFs comes from the same population [38]. For this analysis, the BT and NT tMTF shape distributions were combined into one distribution. We then sampled this distribution with replacement. For each sample, we randomly drew a number of samples that was equal to the number of NT neurons. The proportion of bandpass tMTFs in this random sample was calculated. We repeated this calculation for each random draw, and compiled a distribution of proportions of bandpass tMTFs. By combining the BT and NT shape distributions, this procedure implicitly assumes that there are no differences between BT and NT neurons. To determine significance, the actual proportion of bandpass tMTFs for NT neurons was compared to the compiled resampled distribution. We found that the distributions were significantly different (p = 0.0009, N = 10000 randomizations), indicating that a significantly higher proportion of NT neurons had bandpass tMTFs compared to BT neurons.

The shape of spectral MTFs for BT and NT neurons also differed. NT neurons had a higher proportion of bandpass sMTFs (12%) compared to BT neurons (8.8%). The significant difference in the proportions likely reflects the stronger sideband suppression in the STRFs of NT neurons (p = 0.0493, randomization test).

### Comparison of STRF Parameters and Spectral Tuning

**Figure 9:**
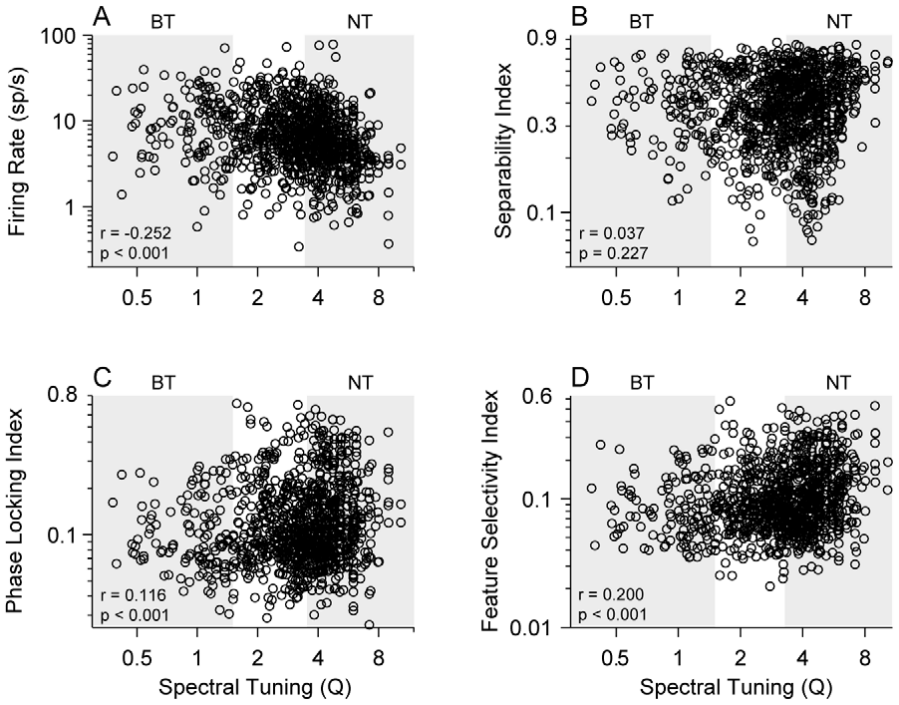
STRF parameters versus spectral tuning (Q). (A) Firing rate is weakly correlated with Q (r = −0.252, p<0.001, t-test). (B) Phase locking index is weakly correlated with Q (r = 0.116, p<0.001, t-test). (C) STRF separability is uncorrelated with Q (r = 0.037, p = 0.227, t-test). (D) Feature selectivity index is weakly correlated with Q (r = 0.200, p<0.001, t-test). Shaded areas Broadly Tuned (BT) and Narrowly Tuned (NT) neurons.

To further evaluate the joint spectrotemporal processing, spectral tuning was compared with firing rate, STRF separability, phase locking to the ripple stimulus, and spectrotemporal feature selectivity. Firing rate decreased with increasing Q, revealing that more broadly tuned neurons have higher discharge rates (Fig. 9A; r = −0.252, p<0.001, t-test; see also above). STRF separability measures the degree to which time and frequency processing may be dissociated in the STRF. If such a dissociation is possible, then the STRF can be approximated by a product of two independent, one-dimensional functions. Separability indices near 1 indicate complete dissociation, while lower values indicate more intricate interactions between time and frequency within the STRF. There was no significant correlation between separability and Q (Fig. 9B; r = 0.037, p = 0.227, t-test; BT median/MAD = 0.61/0.13, NT median/MAD = 0.61/0.14, p>0.25, Rank-sum test). The phase locking index quantifies how precisely the evoked spike responses were time-locked to aspects of the ripple stimulus envelope [27]. Phase locking weakly increased with increasing tuning sharpness (Fig. 9C; r = 0.116, p<0.001, t-test; BT median/MAD = 0.073/0.029, NT median/MAD = 0.094/0.042, p<0.001, Rank-sum test). The feature selectivity index indicates the variability of the individual stimulus segments that elicit a spike and, thus, contributes to the estimation of the STRF. Higher values indicate greater stimulus selectivity [31], [39]. Feature selectivity moderately increased with sharpness of tuning (Fig. 9D; r = 0.200, p<0.001, t-test; BT median/MAD = 0.06/0.02, NT median/MAD = 0.09/0.04). This suggests that a greater range of stimulus segments contribute to BT neuron responsiveness.

### Input/Output Nonlinearities of AI neurons

**Figure 10:**
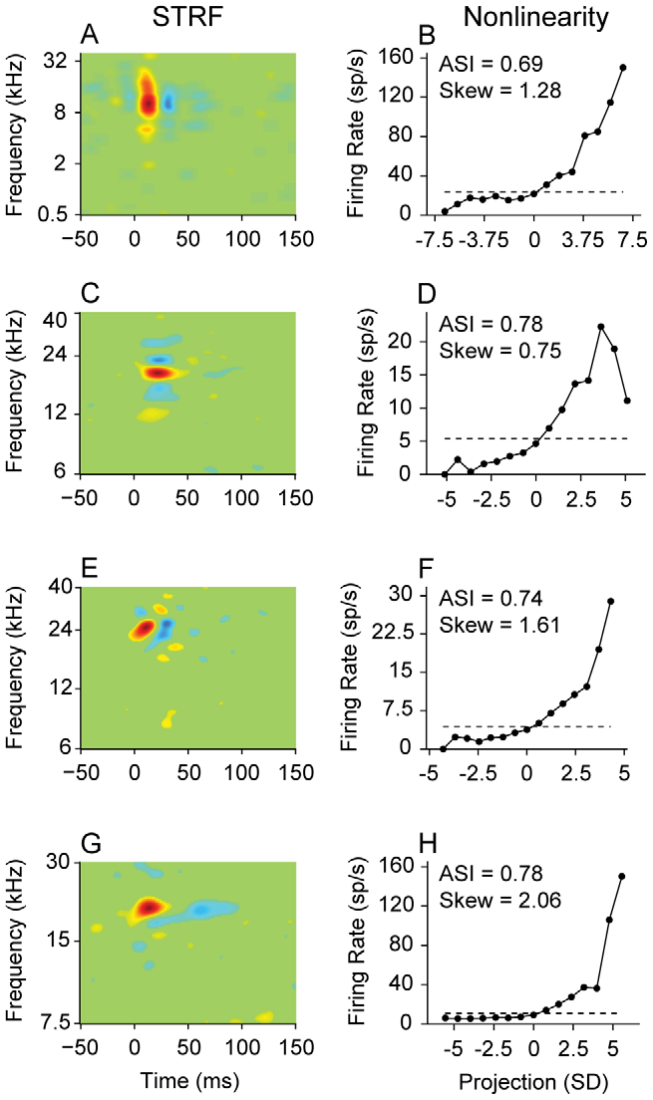
STRF and nonlinearity examples. Each row corresponds to one neuron. (A) BT neuron STRF and (B) corresponding nonlinearity. Dashed line: average firing rate of the neuron during the ripple stimulus. Noted for each neuron are: nonlinearity asymmetry index (ASI; asymmetry of nonlinearity) and nonlinearity skewness (Skew). Firing rate increases as the projection of the stimulus onto the STRF increases (or, equivalently, as the correlation, or similarity, between the stimulus and the STRF increases). (C–H) additional STRF-nonlinearity examples. Abscissas of nonlinearities are in units of standard deviation (SD), where the value indicates the stimulus similarity relative to a randomly selected stimulus pattern. Example: a value of 3 SD represents a similarity value that would on average not be expected for a randomly spiking neuron.

The STRF by itself describes the stimulus features to which a neuron is sensitive and how they may interact. It does not address how the processing of the STRF translates into the firing rate of the neuron. The STRF is one part of a linear-nonlinear (LN) model for a neuron. In the LN framework, the linear filter (STRF) processes stimuli, and the output of the filtering is sent to a nonlinear gain function, or nonlinearity. The nonlinearity describes how the firing rate of a neuron changes as the projection, or similarity, between the stimulus and the STRF changes (Fig. 10B,D,F,H). It contains no time dependence, and may be arbitrarily nonlinear. Nonlinearities may be derived for any neuron with an STRF.

To evaluate nonlinearities, we took two approaches, a non-parametric and a parametric analysis. The first approach was non-parametric, and here we estimated parameters directly from the nonlinearity: the asymmetry index (ASI) and the skewness (Skew). The structure of the nonlinearity may be described by the asymmetry index (ASI). The ASI is defined as ASI = (R−L)/(R+L), where R is the sum of the nonlinearity values for similarity values greater than 0 (positive correlations between stimulus and filter), and L is the sum for similarity values less than 0 (negative correlations between stimulus and filter). The ASI varies between −1 and 1, with 1 corresponding to a nonlinearity where the firing rate increases only when the stimulus is highly similar to the STRF. The skewness of the nonlinearity describes the amount of information in the tail, and thus it describes how quickly the nonlinearity rises with increasing stimulus similarity. Skewness is defined as 
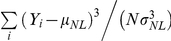
, where *Y_i_* are the nonlinearity values, 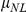 is the mean of the nonlinearity, *N* is the number of nonlinearity points, and 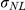 is the standard deviation of the nonlinearity. When the firing rate gradually increases, the skewness will be near 1 (Fig. 10D). When the firing rate increases more rapidly, the skewness values are greater than 1 (Fig. 10F,H).

**Figure 11:**
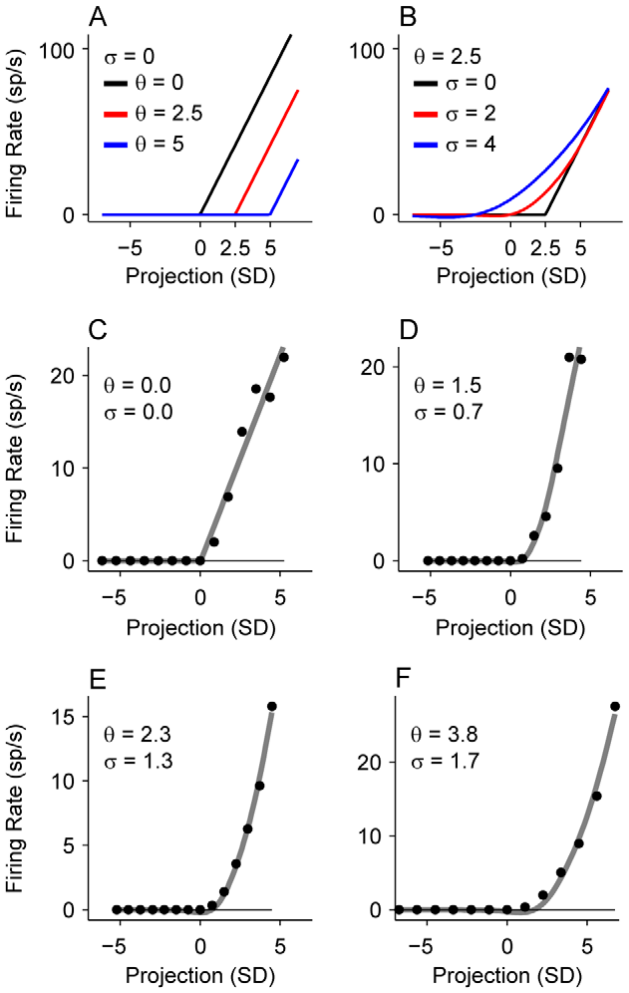
Parametric analysis of STRF nonlinearities. (A) Example parametric curves. In these examples the transition noise σ is set to 0, and the threshold θ is varied. (B) Example parametric curves with the threshold θ held constant at 2.5 and the transition noise σ varied. (C–F) Example nonlinearities from data (black dots) and corresponding parametric curve fits in gray. Threshold and transition values for each curve fit are shown in insets as θ and σ.

In the parametric approach, we used a function that has wide theoretical and experimental support [40], [41]. The function has the following form: 
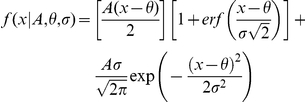
Here, A is a gain term, θ is the threshold to response, and σ is the transition smoothness in the nonlinearity. erf(x) is the error function. We fitted the function to nonlinearity values that exceeded the average firing rate (dashed line in Fig. 10), which indicates responsiveness to the ripple stimulus. For analysis, we only used fits that gave normalized mean squared errors less than 0.1, and coefficient of determination values greater than 0.9. θ and σ are the most significant parameters in the fit: θ determines the threshold, while σ determines how smooth the transition is when the responses become greater than the average firing rate. When σ is 0, the function describes hard rectification. When σ increases, the transition in the nonlinearity smoothly varies (Fig. 11 shows example functions for different values of θ and σ, as well as examples of excellent fits of the function to data nonlinearities).

We analyzed the dependence of these nonlinearity parameters on spectral tuning. The structure, or asymmetry, of the nonlinearity was only weakly correlated with Q (Fig. 11A; r = 0.107, p<0.001). Most ASIs were greater than 0.5, indicating that STRF nonlinearities were highly asymmetrical across the population of AI neurons, i.e., firing rate increased for positive correlations of stimulus and filter. The skewness of the nonlinearity was not significantly correlated with Q (Fig. 11B; r = 0.046, p = 0.134). Therefore, the rate at which the nonlinearity rises is not consistently related to filter bandwidth.

**Figure 12:**
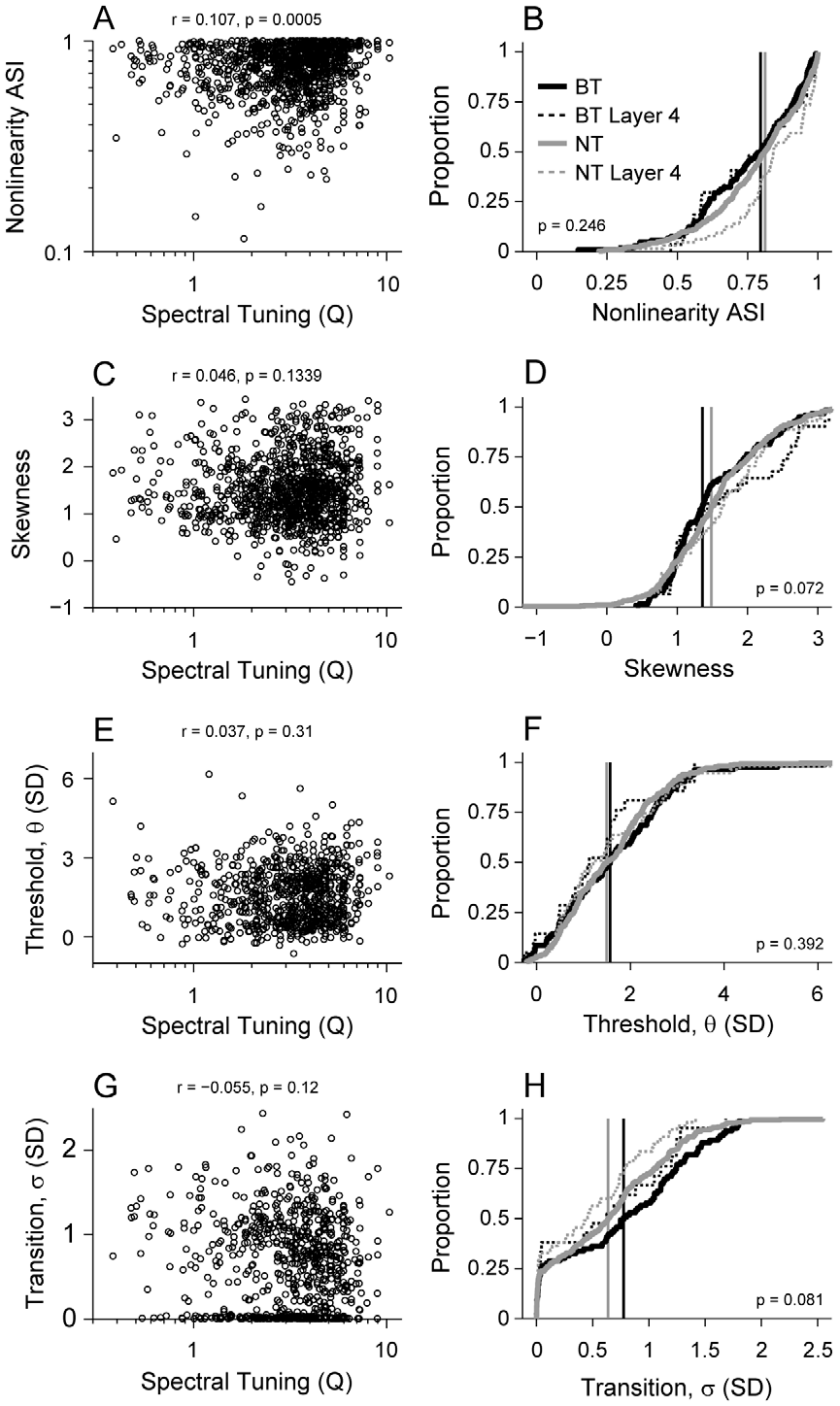
Nonlinearity parameters versus spectral tuning (Q). (A) Nonlinearity asymmetry index (ASI) was weakly correlated with increasing Q (r = 0.107, p<0.001, t-test). (B) BT and NT neurons had similar ASI distributions. Solid lines represent population data. Vertical lines indicate population medians (BT median = 0.79, NT median = 0.81, p = 0.246, KS-test). Dashed lines represent values for neurons in layer 4 (800–1100 µm). (C) Nonlinearity skewness was not significantly correlated with Q (r = 0.046, p = 0.134, t-test). (D) Skewness was significantly different for BT and NT neurons (BT median = 1.36, NT median = 1.49, p = 0.072, KS-test). (E) Nonlinearity threshold, θ, was not correlated with Q (r = 0.037, p = 0.31, t-test). (F) Threshold was similar for BT and NT neurons (BT median = 1.57, NT median = 1.49, p = 0.392, KS-test). (G) Nonlinearity transition noise, σ, was not correlated with Q (r = −0.055, p<0.12, t-test). (H) Nonlinearity transition was similar for BT and NT neurons (BT median = 0.77, NT median = 0.64, p = 0.081, KS-test).

Are nonlinearity parameters related to the topography of primary auditory cortex? Since BT and NT neurons form local, spatial networks within AI, we are implicitly examining the spatial relation of parameters within AI when we separate neurons into these classes. When we parsed our data into BT and NT categories, we found one difference and three similarities for nonlinearity parameters. First, using the asymmetry index, we did not find a significant difference between nonlinearity structure for BT and NT classes (Fig. 12B; BT median/MAD: 0.79/0.14; NT median/MAD: 0.81/0.13; KS-test, p = 0.246). Second, nonlinearity skewness was different for the two spectral tuning classes (Fig. 12D; BT median/MAD: 1.36/0.41; NT median/MAD: 1.49/0.48; KS-test, p = 0.072). Third, we found that the parametric nonlinearity threshold, θ, was similar for BT and NT neurons (Fig. 12F; BT median/MAD: 1.57/0.88; NT median/MAD: 1.49/0.72; KS-test, p = 0.39). Last, the response transition, σ, trended toward higher values for BT neurons, though this difference was not statistically significant (Fig. 12H; BT median/MAD: 0.77/0.55; NT median/MAD: 0.64/0.48; KS-test, p = 0.081). Thus, to a first approximation, the manner in which spikes are generated is similar for BT and NT neurons. Additionally, both NT and BT neurons had equal proportions of neurons with hard rectified responses. The lack of nonlinearity differences between the whole populations was also evident for the input layer neurons alone (Fig. 12 B,D,F,H; dashed lines).

## Discussion

The main result of the current study is that processing modules identified through measures of spectral integration in cat AI show distinct differences with regard to other, stimulus-based response aspects, including spectral and temporal modulation preferences, the shape of modulation filters, envelope phase-locking ability, and stimulus-feature selectivity. This suggests that *stimulus content*, or what stimulus aspect is being processed, is strongly dependent on local circuitry, and shows spatial organization. By contrast, the nonlinearity capturing the input/output rules and the nature of spectrotemporal interactions was found to be similar for NT and BT neurons, suggesting that the *manner* in which stimulus information is expressed in the firing rate is not related to the spectral integration properties of AI neurons.

Our grouping of neurons was based on physiological criteria along a spectral processing dimension. The grouping was confirmed by analyzing the recording location of each neuron, thereby revealing spatial variations in these physiological parameters (Fig. 2). The grouping based on broad-band stimulation corresponded well with estimates using pure-tone tuning curves [6], [7], [8], [9], indicating that the observed phenomenon is a general feature of auditory cortical processing and not specific to the global stimulus design and statistics. This grouping, however, is only one of many possible clustering methods that may be employed. For example, STRFs in the birdsong system have been grouped along spectrotemporal dimensions using various learning rules [42]. Additionally, alternative STRF clustering techniques may be useful for speech analysis and recognition applications [43]. The justification for our grouping, however, is fundamentally different from those approaches. Our motivation for clustering is based on the covariation of anatomical and physiological properties in cat AI. The connectivity pattern of NT regions within AI corresponded to specific, physiologically defined criteria [6]. In this case, spectral integration is used as an assay in which to address the processing within anatomically definable and distinct subregions of cat AI.

### Spectrotemporal processing

Our goal was to identify spectral-temporal processing differences, based on STRFs, between functional circuits dominated by either BT or NT neurons. We exploited the structure of AI by recording within the central narrowly tuned region of AI, and in regions dorsal and ventral to this central region. Previous work has shown that broadly tuned single units are found more commonly in the AI region dorsal, but less so ventral, to the central narrowly tuned region [17]. This physiological boundary was helpful in localizing the topographic location of BT neurons. By using multi-channel recording probes and dynamic moving ripple stimuli, we computed STRFs. The STRF appears to represent the dominant response mode for any neuron, regardless of the complexity of the model [3], [33]. Thus, parameters from the STRFs of BT and NT neurons may be profitably compared (see summary in Table 1). The firing rates of BT neurons were higher than those of NT neurons, possibly due to the larger spectral integration window and shorter time constants of BT neurons. NT neurons had a higher degree of feature selectivity, or stimulus preference, than BT neurons. Best temporal and spectral modulation frequencies covaried with spectral tuning. In contrast to a previous report using pure-tone tuning estimates, we found that spectral tuning derived from broad-band stimuli is proportional to preferred spectral modulation frequency [44].

**Table 1:** Receptive field parameters of Narrowly and Broadly Tuned AI neurons.

	Narrowly Tuned		Broadly Tuned
Firing Rate (sp/s)	3.3 (2.4)	S	7.4 (5.5)
Excitatory Duration (ms)	26.0 (6.5)	S	20.5 (3.4)
Inhibitory Duration (ms)	41.0 (18.5)	S	18.0 (4.5)
Best Temporal Modulation Frequency (cyc/s)	10.5 (3.2)	S	17.3 (4.0)
Best Spectral Modulation Frequency (cyc/oct)	1.19 (0.27)	S	0.21 (0.05)
% Bandpass tMTFs	56	S	37
% Bandpass sMTFs	12	S	9
Phase Locking Index	0.094 (0.042)	S	0.073 (0.029)
STRF Separability	0.612 (0.136)	NS	0.612 (0.129)
Feature Selectivity Index	0.090 (0.041)	S	0.058 (0.024)
Nonlinearity Asymmetry Index	0.81 (0.13)	NS	0.79 (0.14)
Nonlinearity Skewness	1.49 (0.48)	NS	1.36 (0.41)
Nonlinearity Threshold, θ (SD)	1.49 (0.72)	NS	1.57 (0.89)
Nonlinearity Transition, σ (SD)	0.64 (0.48)	NS	0.77 (0.55)

Values represent population medians (median absolute deviations, when applicable, in parentheses). S = significantly different. NS = not significantly different.

The differences in modulation processing for NT and BT neurons point to two possible topographic organizations in AI. First, since best temporal modulation frequency varies with spectral tuning, and spectral tuning displays a topographic organization, it is likely that high and low temporal modulation preference also shows some spatial segregation in AI. Previous studies provided preliminary evidence for this implication, although the relation of spatially non-homogeneous response distributions to single unit responses was not quantified [45], [46]. Our results indicate the possible existence, though not the strength, of spatial segregation of preferred temporal modulation frequencies. However, this organization of temporal information is not likely to take the form of a map given the narrow overall range and fairly low selectivity of temporal tuning. We also found that best spectral modulation frequencies, as derived from the STRFs, was highly correlated with spectral integration width. This provides further evidence that a spatial segregation of spectral modulation preferences coexists with a spectral decomposition domain – the tonotopic axis - in AI [47]. Since spectral tuning and spectral modulation are highly correlated and span a fairly broad parameter range, we expect at least a rudimentary map of spectral modulation in AI. Overall, the findings support the notion that AI modules specialize in processing either narrowband, slowly changing or broadband, more quickly changing stimuli.

### Input/Output Nonlinearities

The nonlinear input/output functions for NT and BT neurons were strikingly similar, despite the large difference in preferences for spectral and/or temporal stimulus parameters. The nonlinearity describes how the spectrotemporal processing of a neuron is translated into an output firing rate. That the structure of the nonlinearities, including asymmetry and skewness, was not significantly different indicates that the spectral integration modules translate processing to output in similar ways across AI circuits. Additionally, the similar nonlinearity thresholds between the neuronal groups indicate that the operating point of AI neurons is independent of stimulus content or potential local circuit differences. The distribution of thresholds was centered at approximately 1.5 SD (Fig. 12). This value represents the strength of the similarity between stimulus and filter that is required to drive a neuron to spike. Since the value is shifted away from 0 SD, it implies that the stimulus-filter similarity must be removed from the mean, stochastic similarity by a significant amount for discernible firing rate responses to ensue. This process, of gating or suppressing responses until significant stimulus similarity is achieved, is appropriate for providing stimulus selectivity, eliminating spurious matches from noise, and enhancing the ability to detect signals in masking conditions. The value of 1.5 SD is higher than that reported for visual cortex by Ringach and Malone (2007) [48], from which we appropriated the parametric approach. Further, the nonlinearity transition smoothness in AI is approximately 0.7 SDs, similar to that in visual cortex. This would imply that although the spiking response transition may be similar across the different sensory modalities, auditory cortical processing is more specialized to emphasize large signal deviations from random stimulus-filter matches, while suppressing those that do not reach appropriate levels of salience.

### Connectivity

The connection patterns between spectral tuning modules are most similar to those in visual cortex for orientation tuning [49]. Tracer injections into regions sensitive to orientations in V1 also revealed patchy labeling more than 1.0 mm from the injection site, showing that local functional networks are present with many of the same characteristics as those in AI [50]. Additionally, V1 cross-correlation studies showed that these regions are functionally connected [5]. Though the patchy labeling in AI and V1 is similar, studies have not described the feature selectivity and temporal response characteristics between connected regions in VI. Thus, we do not know if complex stimulus processing is similar in these two systems.

### Future work and implications

Besides AI, other cortical fields, especially the anterior auditory field in the cat, also show bandwidth modules [51]. However, the modules in these fields are smaller than in AI and their location within the tonotopic map is more variable. Other species such as squirrel monkey and owl monkey, also exhibit local clusters of BT and NT neurons in AI, though again their location and extent is more variable than in cat AI [17], [52], [53]. This might indicate that an organization principle based on spectral integration could be widespread in mammalian core areas, though evidence for spectral integration modules in some other species is either weak (rat; [54]) or absent (ferret; [55]).

Our study suggests several directions for future work. Specifically, we need to examine how STRFs in the spectral integration modules vary with layer. Previous studies have indicated clear layer differences [1], [3], although their relationship to modular organization and inter-module differences is still unresolved. Additionally, since multiple NT and BT modules exist in AI, we need to examine how the connectivity between similar modules contributes to spectrotemporal processing. Thus, while anatomical studies showed that there are at least two subregions related to spectral tuning, our work combined all neurons according to spectral tuning, regardless of anatomical location. To further dissect the function of these networks in AI, we need to determine, for example, how the processing in one narrowly tuned region affects the other. The spatial resolution of the experiments required for this analysis would be challenging though approachable.

Since NT and BT neurons exhibit fundamental receptive field differences, it is also probable that the spectral integration modules are members of separate functional streams in AI [56], [57]. Here, the concept of streams is based on anatomical and physiological evidence and not on a conceptual picture of auditory perception. In the latter case, the idea of functional streams is related to the processing of sound identification and sound location, which are perceptual entities without a well-understood biological basis [58], [59]. Indeed, sound identification has been formulated as either pitch perception or complex sound or object identity, both of which are not well understood at the single neuron level. Further, a topographic map of auditory space in AI has not yet been identified, and thus location information may be distributed throughout auditory cortex [60], [61], [62]. In the present study, we identified spectral integration streams from single neuron STRF properties that are related to anatomical differences. To determine if these streams are fundamental to auditory processing, they need to be traced from AI to later fields, such as the posterior or ventral-posterior auditory fields [63], [64]. It is likely that the segregated NT and BT information in AI is also distributed differently at the next stage of the auditory cortex hierarchy, where it may be processed and combined to subserve specific behavioral demands. That spectral integration information is segregated, in AI and in other fields [51], is consistent with the highly topographic projection patterns between all auditory cortical fields [65].

Our results have implications for studies of forward masking and signal detection in noise in AI [66], [67]. With regard to forward masking, since the excitatory and inhibitory subfields of NT and BT neurons differ, it is likely that masking will also vary between the different modules in AI. The central region of AI would be expected to show a longer time course of forward masking due to the greater duration of inhibition in NT STRFs [67]. Alternatively, BT neurons are expected to display an increased proclivity to recover from the effects of sequential acoustic stimulation. Further, since the bandwidths of the excitatory and inhibitory subfields are not matched for BT neurons, the frequency of the masker will also be differentially represented in the responses of BT neurons [68]. Thus, the NT and BT networks should exhibit clear differences when classical stimulation protocols are applied.

With regard to signal detection in noise, our results imply that the different bandwidth modules serve different functions. The central narrowly tuned region, due to the prevalence of sharply tuned filters, is more likely to be involved in signal reconstruction when stimuli are embedded in noise [69]. The necessity of sharply tuned filters for detecting signals in noise is reflected in the correlation between signal detection and spectral ripple processing in normal and hearing impaired subjects [70], [71], [72], [73]. High spectral modulation following abilities correspond to increased abilities in detecting speech in background noise. This is accomplished through the narrower tuning of auditory filters. A direct test of this phenomenon would involve behavioral performance of cats before and after inactivating the central NT region. We expect that signal detection thresholds will rise when the NT region is inactivated.

We conclude by noting that our approach employed multi-channel neuronal recordings combined with dynamic stimulation, allowing us to reconstruct complete spectrotemporal STRFs for large populations of neurons in AI spectral tuning modules. In the future, this approach can be adapted to analyze neurons using information theoretic approaches that may reveal additional spectrotemporal dimensions that contribute to the response behavior of cortical neurons [33]. These can then help determine the temporal precision of groups of neurons, or to determine the stimulus dimensions, and accompanying nonlinearities, that best describe a neuron's spiking response [74], [75].
